# Maternal iron status in early pregnancy and DNA methylation in offspring: an epigenome-wide meta-analysis

**DOI:** 10.1186/s13148-022-01276-w

**Published:** 2022-05-03

**Authors:** M. J. Taeubert, P. de Prado-Bert, M. L. Geurtsen, G. Mancano, M. J. Vermeulen, I. K. M. Reiss, D. Caramaschi, J. Sunyer, G. C. Sharp, J. Julvez, M. U. Muckenthaler, J. F. Felix

**Affiliations:** 1 The Generation R Study Group, Erasmus University Medical Center, PO Box 2040, 3000 CA Rotterdam, The Netherlands; 2 Department of Pediatric Oncology, Hematology and Immunology, University Medical Center Heidelberg, Heidelberg, Germany; 3 ISGlobal, Barcelona, Spain; 4 Universitat Pompeu Fabra (UPF), Barcelona, Spain; 5 CIBER Epidemiología y Salud Pública (CIBERESP), Madrid, Spain; 6 Department of Pediatrics, Sophia’s Children’s Hospital, Erasmus MC, University Medical Center Rotterdam, Rotterdam, the Netherlands; 7 College of Life and Environmental Sciences, Psychology, University of Exeter, Exeter, UK; 8 MRC Integrative Epidemiology Unit, University of Bristol, Bristol, UK; 9 Bristol Medical School Population Health Sciences, University of Bristol, Bristol, UK; 10 IMIM (Hospital del Mar Medical Research Institute), Barcelona, Spain; 11 School of Oral and Dental Sciences, University of Bristol, Bristol, UK; 12 Institut d’Investigació Sanitària Pere Virgili, Hospital Universitari Sant Joan de Reus, Reus, Spain

**Keywords:** DNA methylation, Iron metabolism, Differentially methylated regions, Epigenetics, Maternal serum ferritin

## Abstract

**Background:**

Unbalanced iron homeostasis in pregnancy is associated with an increased risk of adverse birth and childhood health outcomes. DNA methylation has been suggested as a potential underlying mechanism linking environmental exposures such as micronutrient status during pregnancy with offspring health. We performed a meta-analysis on the association of maternal early-pregnancy serum ferritin concentrations, as a marker of body iron stores, and cord blood DNA methylation. We included 1286 mother–newborn pairs from two population-based prospective cohorts. Serum ferritin concentrations were measured in early pregnancy. DNA methylation was measured with the Infinium HumanMethylation450 BeadChip (Illumina). We examined epigenome-wide associations of maternal early-pregnancy serum ferritin and cord blood DNA methylation using robust linear regression analyses, with adjustment for confounders and performed fixed-effects meta-analyses. We additionally examined whether associations of any CpGs identified in cord blood persisted in the peripheral blood of older children and explored associations with other markers of maternal iron status. We also examined whether similar findings were present in the association of cord blood serum ferritin concentrations with cord blood DNA methylation.

**Results:**

Maternal early-pregnancy serum ferritin concentrations were inversely associated with DNA methylation at two CpGs (cg02806645 and cg06322988) in *PRR23A* and one CpG (cg04468817) in *PRSS22*. Associations at two of these CpG sites persisted at each of the follow-up time points in childhood. Cord blood serum ferritin concentrations were not associated with cord blood DNA methylation levels at the three identified CpGs.

**Conclusion:**

Maternal early-pregnancy serum ferritin concentrations were associated with lower cord blood DNA methylation levels at three CpGs and these associations partly persisted in older children. Further studies are needed to uncover the role of these CpGs in the underlying mechanisms of the associations of maternal iron status and offspring health outcomes.

**Supplementary Information:**

The online version contains supplementary material available at 10.1186/s13148-022-01276-w.

## Background

Iron is an essential micronutrient and a critical cofactor for proteins involved in fundamental processes such as oxygen transport, energy production, and DNA synthesis [1]. Iron concentrations need to be tightly balanced to avoid pathological consequences of iron deficiency or overload [1, 2]. European studies have reported a prevalence of iron deficiency ranging from 10 to 33% among premenopausal women, whereas iron overload is less common with a prevalence of around 3% [3]. Multiple environmental, physiological, pathologic, and genetic factors may influence iron concentrations. The most prominent factors contributing to iron deficiency are a decreased iron intake, which can be related to socioeconomic factors, chronic blood loss, and increased iron demand in growing children as well as pregnant women [4]. Iron overload is more commonly caused by hemochromatosis [5].

In pregnancy, both iron deficiency and iron overload have been associated with an increased risk of adverse birth outcomes such as preterm birth, low birth weight (LBW), and small for gestational age (SGA) infants [6, 7]. Furthermore, a link between iron metabolism during pregnancy and offspring neurodevelopment and cognitive function [8] has been described [9–12]. The underlying biological mechanisms for these associations remain to be elucidated.

DNA methylation has been suggested as a potential mechanism linking environmental exposures such as micronutrient status during pregnancy and offspring health [13]. Aberrant iron concentrations are associated with oxidative stress, which in turn has been reported to lead to a lower activity of the ten-eleven translocation (TET) enzyme, an enzyme involved in DNA demethylation [14]. Studies performed in mouse and pig models have linked neonatal iron deficiency with differential DNA methylation in the hippocampus [15, 16]. In humans, little is known regarding the associations of iron status and DNA methylation. The few studies available differ strongly in study design, study population, exposure measurement, and assessment of DNA methylation and show inconsistent results with no clear pattern of association [17–20]. To the best of our knowledge, only one study has previously analyzed the association of maternal iron intake in pregnancy and offspring DNA methylation [20]. In this prospective pre-birth cohort study comprising 516 mother–infant pairs, Boeke et al. found that gestational dietary iron intake, estimated through food frequency questionnaires, was not associated with global LINE-1 DNA methylation in offspring [20]. It remains to be examined whether iron status determined from blood in early pregnancy is associated with DNA methylation in offspring at birth. Deepening the knowledge of these associations is important to better understand the potential underlying mechanisms for the pathologies observed in offspring when maternal iron status in pregnancy is impaired.

We hypothesized that maternal iron status in early pregnancy is associated with offspring DNA methylation at birth. Therefore, we performed a meta-analysis on the associations of maternal serum ferritin concentrations, as a reliable measure of body iron stores, measured in early pregnancy with epigenome-wide offspring DNA methylation. For any significant findings, we examined the associations of maternal serum ferritin concentrations with child peripheral blood DNA methylation, in order to check whether the associations persist over time. Next to this, we assessed whether potential associations with offspring DNA methylation are specific to maternal serum ferritin concentrations or whether there are also associations of cord blood serum ferritin with cord blood DNA methylation.

## Results

### Participant characteristics

A total of 1286 mother–newborn pairs were included in the meta-analysis examining the associations of maternal serum ferritin concentrations with cord blood DNA methylation (*n* = 910 and *n* = 376 mother–newborn pairs from the Generation R Study and the Proyecto Infancia y Medio Ambiente (INMA), respectively). Maternal and child characteristics of the participants are shown in Table 1. The median maternal early-pregnancy serum ferritin concentration was 61.8 µg/L (95% range 13.0, 208.2) and 27.79 µg/L (95% range 5.1, 106.2) in the Generation R Study and the INMA Study, respectively. The participants were all of European ancestry, and approximately half of the newborns were male (*n* = 638 [49.6%]).

**Table 1:** Maternal and child characteristics

	Generation R Study	INMA
	*n* = 910	*n* = 376
*Maternal characteristics*		
Age, years	31.5 ± 4.1	30.4 ± 4.1
Pre-pregnancy body mass index, kg/m^2^	23.2 ± 3.8	23.8 ± 4.5
Gestational age at serum ferritin measurement, weeks	12.8 (9.9, 17.0)	13.2 (11.0, 17.2)
Education, higher	597 (65.6)	115 (30.6)
Continued smoking during pregnancy	114 (12.5)	110 (29.3)
Serum ferritin, µg/L	61.8 (13.0, 208.2)	27.8 (5.1, 106.2)
Iron deficiency (ferritin < 15 µg/L)	31 (3.4)	86 (22.9)
Iron overload (ferritin > 150 µg/L)	85 (9.3)	2 (0.5)
*Child characteristics*		
Sex, male	445 (48.9)	193 (51.3)
Gestational age at birth, weeks	40.4 (36.7, 42.3)	39.9 (36.9, 42.0)
Birth weight, grams	3552 ± 505	3272 ± 419

Values are means ± SD, medians (95% range) or numbers of subjects (valid %)

### Associations of maternal early-pregnancy serum ferritin concentrations with single-CpG DNA methylation at birth

**Fig. 1:**
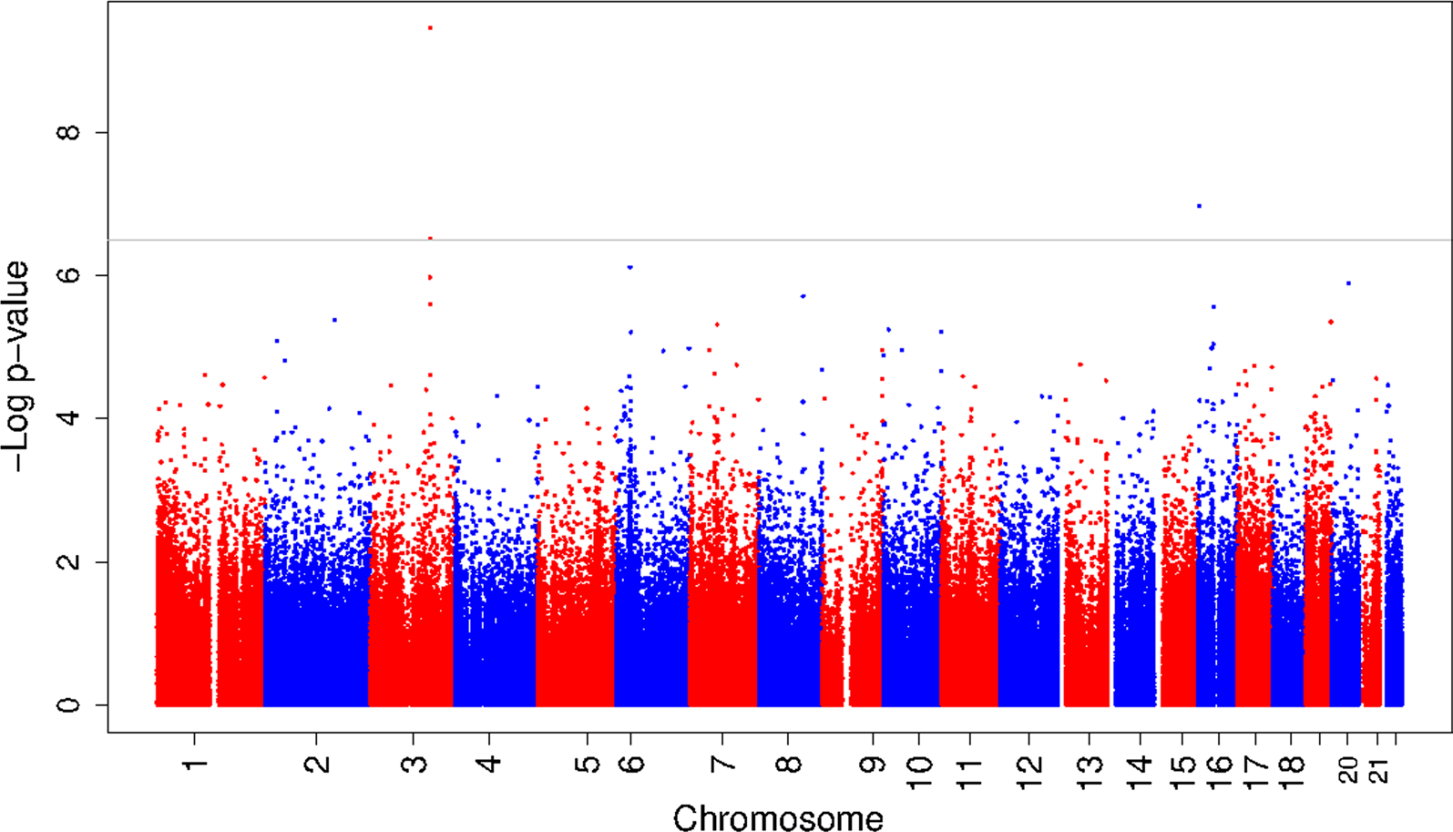
Results of the meta-analysis of epigenome-wide association study results of maternal early-pregnancy serum ferritin concentrations and DNA methylation in cord blood. The Manhattan plot shows the results of the meta-analysis of epigenome-wide association studies of maternal early-pregnancy serum ferritin concentrations and DNA methylation in cord blood. The *x*-axis represents the autosomal (1–22) chromosomes, and the y-axis shows the − log_10_ (*p* value). The gray line indicates the FDR-adjusted significance cutoff. The models were adjusted for gestational age at serum ferritin measurement, maternal age at intake, educational level, pre-pregnancy body mass index, smoking, child sex, cell-type proportions, and batch (model 3)

We used robust linear regression models to examine the associations of early-pregnancy serum ferritin with offspring DNA methylation in cord blood. The main model (model 3) was adjusted for gestational age at serum ferritin measurement, maternal age at intake, educational level, pre-pregnancy body mass index, smoking, child sex, cell-type proportions, and batch. The genomic inflation factors (λ) for the individual cohort results were 1.05 and 1.03 for the Generation R Study and INMA, respectively. We meta-analyzed the results of the individual cohorts using METAL. The λ for the meta-analysis was 1.06. Maternal early-pregnancy serum ferritin concentrations were associated with DNA methylation at three CpG sites after false discovery rate (FDR) correction (cg02806645 in *PRR23A*; effect estimate = -2.5 × 10^−4^ (standard error (SE) 4.0 × 10^−5^) per unit increase in serum ferritin concentration, p value = 3.4 × 10^−10^, FDR = 1.7 × 10^−4^, cg04468817 in *PRSS22*; effect estimate = -1.3 × 10^−4^ (SE 2.4 × 10^−5^), p value = 1.1 × 10^−7^, FDR = 2.5 × 10^−2^, and cg06322988 in *PRR23A*; effect estimate = -1.1 × 10^−4^ (SE 2.1 × 10^−5^), p value = 3.0 × 10^−7^, FDR = 4.9 × 10^−2^) (Fig. 1 and Additional file 2: Table S1). The direction of effect was consistent between the cohorts for these three CpG sites, and there was no evidence of heterogeneity (all *I*^2^ values ≤ 28.3). Results from models 1 (adjusted for gestational age at serum ferritin measurement, child sex, and batch) and 2 (adjusted for gestational age at serum ferritin measurement, child sex, batch, maternal age at intake, educational level, pre-pregnancy body mass index (BMI), and smoking) can be found in Additional file 2: Tables S2 and S3.

### Differentially methylated regions

Using the dmrff package in R, we identified differentially methylated regions (DMRs) in relation to maternal early-pregnancy serum ferritin concentrations. Two regions were differentially methylated in association with maternal early-pregnancy serum ferritin concentrations: one on chromosome 3: 138,725,153–138,725,311 (*PRR23A*, effect estimate =  − 2.7 × 10^−3^ (SE 4.4 × 10^−4^), *p* value = 4.0 × 10^−10^) and one on chromosome 16: 2,902,921–2,903,392 (*PRSS22*, effect estimate =  − 2.2 × 10^−3^ (SE 3.9 × 10^−4^), *p* value = 1.7 × 10^−8^) (Table 2). Both regions showed lower DNA methylation with increasing serum ferritin concentrations.

**Table 2:** Differentially methylated regions associated with maternal early-pregnancy serum ferritin concentrations*

DMR	CpGs	Effect	SE	*p* value	Nearest genes	Gene Group
chr 3: 138,725,153–138,725,311	cg02806645	− 2.7 × 10^−3^	4.4 × 10^−4^	4.0 × 10^−10^	*PRR23A*	*TSS200* *TSS1500*
	cg06322988					
	cg01926813					
	cg07452777					
	cg03772909					
chr 16: 2,902,921–2,903,392	cg06186790	− 2.2 × 10^−3^	3.9 × 10^−4^	1.7 × 10^−8^	*PRSS22*	*3'UTR* *Body*
	cg16701914					
	cg06077305					
	cg04468817					

Chr, Chromosome; DMR, differentially methylated region; TSS, transcription start site; UTR, untranslated region; SE, standard error

*DMRs were identified from the results of the meta-analysis of epigenome-wide association study results of maternal early-pregnancy serum ferritin concentrations and DNA methylation in cord blood for individual CpGs, which used results from robust linear regression models that were adjusted for gestational age at serum measurement, maternal age at intake, educational level, pre-pregnancy body mass index, smoking, child sex, cell-type proportions, and batch (model 3)

### Additional analyses

Results for the identified CpG sites did not substantially change after additionally adjusting the main model for gestational age at birth or birth weight (Additional file 1: Table S4). In addition, results were similar when we performed a sensitivity analysis excluding all mothers with acute inflammation (CRP > 10 mg/L) (Additional file 2: Table S5). After additionally adjusting the main model for Mediterranean diet score or the first genetic principal component, the effect estimates for the three CpGs changed only minimally, but two CpGs (cg06322988 and cg04468817) and one CpG (cg06322988), respectively, did not reach statistical significance anymore (Additional file 1: Table S6).

### Look-up analyses

Maternal early-pregnancy serum ferritin concentrations were also associated with DNA methylation levels at cg02806645 (*PRR23A*) and cg06322988 (*PRR23A*) in early childhood (*n* = 514) and at cg02806645 (*PRR23A*) and at cg04468817 (*PRSS22*) in late childhood (*n* = 490) (Table 3). These models were adjusted for the same covariates as the main model, with additional adjustment for child age and using the Houseman reference for estimating six white blood cell-type proportions instead of the cord blood-specific reference. Finally, within a sample of 311 children in the ALSPAC study (Additional file 1: Table S7), cord blood serum ferritin concentrations were not associated with cord blood DNA methylation levels at the three identified CpGs (Table 4).

**Table 3:** Look-up of three CpGs identified in cord blood in meta-analysis of epigenome-wide association study results of maternal early-pregnancy serum ferritin concentrations and DNA methylation in older children

CpG	Chr	Position	Gene	Effect	SE	*p* value	*I*^2^
*a. Early childhood*							
cg02806645	3	138,725,153	*PRR23A*	− 1.9 × 10^–4^	5.8 × 10^–5^	7.3 × 10^–4^	0
cg06322988	3	138,725,189	*PRR23A*	− 9.7 × 10^–5^	3.0 × 10^–5^	1.4 × 10^–3^	0
cg04468817	16	2,903,392	*PRSS22*	− 8.4 × 10^–5^	4.1 × 10^–5^	3.8 × 10^–2^	0
*b. Late childhood*							
cg02806645	3	138,725,153	*PRR23A*	− 2.8 × 10^–4^	6.3 × 10^–5^	9.7 × 10^–6^	0
cg06322988	3	138,725,189	*PRR23A*	− 5.7 × 10^–5^	2.8 × 10^–5^	4.2 × 10^–2^	77
cg04468817	16	2,903,392	*PRSS22*	− 1.2 × 10^–4^	4.0 × 10^–5^	2.2 × 10^–3^	0

Chr, Chromosome; SE, standard error; *I*^2^, *I*-square

Effect estimates represent the difference in DNA methylation per 1 µg/L increase in maternal early-pregnancy serum ferritin concentrations. The model was adjusted for gestational age at serum ferritin measurement, maternal age at intake, educational level, pre-pregnancy body mass index, smoking, child sex, child age, cell-type proportions, and batch

**Table 4:** Associations of DNA methylation at the three CpGs identified in the analysis of cord blood serum ferritin concentrations and cord blood DNA methylation

CpG	Chr	Position	Gene	Effect	SE	*p* value
cg02806645	3	138,725,153	*PRR23A*	− 1.4 × 10^–5^	5.2 × 10^–5^	0.79
cg06322988	3	138,725,189	*PRR23A*	− 1.9 × 10^–6^	2.2 × 10^–5^	0.93
cg04468817	16	2,903,392	*PRSS22*	− 3.3 × 10^–5^	2.6 × 10^–5^	0.20

Chr, Chromosome; SE, standard error

Effect estimates represent the difference in DNA methylation per 1 µg/L increase in cord blood serum ferritin concentrations. The model was adjusted for gestational age at birth, maternal age at intake, educational level, pre-pregnancy body mass index, smoking, child sex, cell-type proportions, and batch

### Exploratory analyses of associations of maternal early-pregnancy TSAT, serum iron, and transferrin concentrations with DNA methylation at birth

To explore associations of further markers of maternal iron metabolism with offspring DNA methylation, we assessed the associations of maternal early-pregnancy TSAT, serum iron, and transferrin concentrations with offspring DNA methylation, at a single CpG and DMR level, within the Generation R Study using robust linear regression models. These models were adjusted for the same covariates as the main serum ferritin model. Maternal early-pregnancy transferrin concentrations were associated with DNA methylation at one CpG (cg09996156 in *KIAA1324L*; effect estimate =  − 5.5 × 10^−3^ (SE 1.0 × 10^−3^), *p* value = 4.7 × 10^−8^) after FDR correction (Additional file 2: Table S8). We did not observe associations of maternal early-pregnancy TSAT or serum iron concentrations with offspring single-CpG DNA methylation in cord blood (Additional file 2: Tables S9 and S10).

Maternal early-pregnancy TSAT was associated with differential DNA methylation in one region (chromosome 16: 67,225,165–67,225,924, *E2F4*, effect estimate = 1.0 × 10^−4^ (SE 1.9 × 10^−5^), *p* value = 3.7 × 10^−8^) after FDR correction (Additional file 1: Table S11). We did not observe associations of maternal early-pregnancy serum iron or transferrin concentrations with offspring regional DNA methylation in cord blood.

In a look-up analysis of the identified CpGs from the serum ferritin meta-analysis, maternal early-pregnancy transferrin concentrations were associated with DNA methylation at cg02806645 (*PRR23A*) and cg06322988 (*PRR23A*), but not at cg04468817 (*PRSS22*). TSAT and serum iron concentrations were not associated with DNA methylation at any of the three CpG sites identified (Additional file 1: Table S12).

### Functional analyses

Pathway and gene ontology enrichment analyses for the main serum ferritin results did not reveal any significantly enriched biological pathways or processes and no tissue-specific enrichment was observed. In addition, the three CpGs identified in association with serum ferritin were not significantly associated with gene expression in a publicly available eQTM database from child blood. A look-up of the CpGs identified in association with serum ferritin in the UCSC Genome Browser showed that the three CpGs were situated within DNAseI hypersensitive sites, which are associated with transcriptional activity. Using the Blood Brain DNA Methylation Comparison Tool, we observed that methylation levels at the three CpGs in blood correlated moderately with brain methylation levels in different brain regions (correlation coefficients 0.25–0.57) (Additional file 1: Table S13) [21]. Next, we performed a look-up in a mouse-knockout database of the genes annotated to the differentially methylated CpGs and regions in the primary serum ferritin analysis and in the exploratory analyses of the additional iron markers. Mice in which *KIAA1324L* was knocked out displayed abnormalities of the hematopoietic system such as decreased mean platelet volume and decreased leukocyte cell number [22]. *E2F4* knockout mice showed a broad range of phenotypes, including aberrant hematopoietic lineage development, leading to anemia [23, 24]. No mouse knockout information was reported for *PRR23A* or *PRSS22*. Finally, a look-up in results from EWASs of neurodevelopmental outcomes including ADHD symptoms, autism spectrum disorder (ASD), and IQ showed that DNA methylation at the three identified CpGs was not associated with these outcomes (Additional file 1: Table S14) [25–27].

## Discussion

In this population-based epigenome-wide meta-analysis on the associations of maternal early-pregnancy serum ferritin concentrations with DNA methylation in the offspring, we observed that serum ferritin concentrations were associated with DNA methylation at three CpG sites. Associations at two of these CpG sites persisted at each of the follow-up time points in early (4- and 6-year-old children) and late childhood (9- and 10-year-old children). Maternal early-pregnancy serum ferritin concentrations were also associated with differential methylation in two regions. However, cord blood serum ferritin concentrations were not associated with cord blood DNA methylation levels at the three identified CpGs.

### Interpretation of main findings

A dysregulated iron metabolism in pregnancy has been associated with an increased risk of various adverse birth outcomes [6, 7], as well as with impaired offspring neurodevelopmental outcomes such as cognitive dysfunction [8], autism, and schizophrenia [9, 10]. An analysis of ferritin concentrations as a continuous measure showed an inverse association with inattention [11]. In addition, maternal serum ferritin concentrations in the normal range have been reported to be associated with better working memory and executive functioning in the offspring [12]. Differential DNA methylation may play a role in the associations of iron metabolism in pregnancy with offspring health outcomes. Iron concentrations may affect DNA methylation through oxidative stress. Both low and high iron concentrations can cause increased oxidative stress, and oxidative stress has been reported to lead to a lower activity of the ten-eleven translocation (TET) enzyme which is involved in DNA demethylation [14]. In addition, hypoxia-related mechanisms may play a role [28, 29]. Therefore, we hypothesized that maternal iron status in early pregnancy is associated with offspring DNA methylation at birth.

To the best of our knowledge, no previous study has investigated associations of maternal iron status in early pregnancy with single-CpG and regional epigenome-wide DNA methylation in cord blood. In the current meta-analysis, we observed that maternal early-pregnancy serum ferritin concentrations were associated with decreased DNA methylation at cg02806645 (*PRR23A*), cg06322988 (*PRR23A*), and cg04468817 (*PRSS22*). We also identified two differentially methylated regions within the *PRR23A* and *PRSS22* genes in relation to serum ferritin concentrations, strengthening the associations of maternal early-pregnancy serum ferritin concentrations with offspring DNA methylation in these genomic regions.

*PRR23A*, Proline-Rich Protein 23A, is located on chromosome 3 and belongs to the PRR23 family of proteins. *PRR23A* is expressed the strongest in the testis, ovaries, brain, and heart. Its function is not clear, but genetic variants in this gene have been previously associated with androgenic alopecia, keloids, and brain morphology [30–34]. *PRSS22*, serine protease 22, is located on chromosome 16 and is part of the trypsin family of serine proteases, enzymes that cleave peptide bonds in proteins. *PRSS22* is expressed in most tissues, including secretory and internal organs. In addition, this gene appears to be expressed in the airways in a developmentally regulated manner [35]. No studies have been previously reported that assessed the link between the *PRR23A* and *PRSS22* genes and iron metabolism. The three CpGs identified in this study in association with maternal serum ferritin concentrations were not found to be associated with ADHD symptoms, autism spectrum disorder (ASD), or IQ in previous EWASs [25–27]. This may suggest that DNA methylation at these CpGs does not mediate the associations of iron metabolism with these specific neurodevelopmental outcomes, but these analyses should be interpreted as exploratory.

Maternal serum ferritin concentrations in early pregnancy were associated with DNA methylation in the peripheral blood of older children at two of the CpG sites identified in cord blood at each of the follow-up time points. This suggests a partly persistent effect of maternal early-pregnancy serum ferritin concentrations on offspring DNA methylation. In contrast, cord blood serum ferritin concentrations were not associated with cord blood DNA methylation levels at the three identified CpGs. A recent study reported a weak correlation between maternal serum ferritin and newborn serum ferritin concentrations of 0.14 (CI: 0.07, 0.20; *p* value < 0.0001) [36]. The lack of association in cord blood may indicate that early pregnancy is a critical period for potential effects of unbalanced iron levels on offspring DNA methylation. Alternatively, we may have lacked statistical power to detect the associations due to a smaller sample size in the analysis of cord blood ferritin concentrations. Therefore, further studies with a larger sample size are needed to confirm these findings. In additional analyses, we included Mediterranean diet score and genetic PC1 in the main model of the serum ferritin meta-analysis. The effect estimates were very consistent, but two CpGs and one CpG, respectively, did not reach statistical significance anymore. Although this may represent some residual confounding by diet and ethnicity, we also consider it likely that the lower sample size combined with an additional covariate in the models led to a lower of power for these analyses.

Within the Generation R Study, we additionally examined associations of other markers of iron metabolism in early pregnancy with offspring DNA methylation. Maternal early-pregnancy TSAT was associated with differential DNA methylation at one region within the *E2F4* gene. *E2F4*, E2F Transcription Factor 4, plays a vital role in the control of cell cycle and is expressed in many tissues. Maternal early-pregnancy transferrin concentrations were associated with increased DNA methylation at cg09996156 (*KIAA1324L*). *KIAA1324L*, Endosome-Lysosome Associated Apoptosis And Autophagy Regulator Family Member 2, functions as a regulator of the Bone Morphogenetic Protein (BMP) pathway, which is crucial in embryogenesis and development [37]. This gene is most strongly expressed in the lung and brain and has been associated with nervous system development. Interestingly, mouse knockouts of both *E2F4* and *KIAA1324L* display abnormalities in the hematopoietic system [22–24]. Finally, in a look-up analysis of the three CpGs identified in the serum ferritin meta-analysis, maternal early-pregnancy transferrin concentrations were associated with increased DNA methylation at the two CpG sites identified within the *PRR23A* gene. In an iron-deficient state, iron stores are increasingly mobilized by transferrin in order to supply cells with iron. Therefore, serum ferritin and transferrin concentrations are inversely correlated. This could explain the opposite directions of effect of the associations of serum ferritin and transferrin with DNA methylation at the identified CpGs. The overlapping effects indicate that differential methylation at these CpG sites may be related to multiple components of iron metabolism.

Our results suggest the association of maternal serum ferritin concentrations with differential DNA methylation at three CpGs sites in offspring. Future studies should be performed to confirm these findings and to assess whether these associations represent an underlying biological mechanism linking maternal iron metabolism and offspring health outcomes.

### Methodological considerations

A major strength of this study is the population-based prospective design of the participating cohorts. In addition, we were able to increase the statistical power of the study by meta-analyzing results from multiple cohorts and the models were adjusted for a large number of potential confounders and for estimated cell-type proportions. Next to single-CpG analyses, differentially methylated regions were also evaluated. Within the Generation R Study, we also had information on multiple markers of iron status in early pregnancy, allowing us to explore the effects of iron bioavailability in the body. Nevertheless, the results of our study should be interpreted in the context of its limitations. First, blood samples were taken in a non-fasting state in the Generation R Study, which could potentially affect the reliability of TSAT and serum iron concentrations due to recent intake of iron-containing foods. Future studies using fasting samples to determine these iron markers are needed to confirm those findings. As serum ferritin concentrations reflect body iron stores, we expect these not to be strongly affected by recent food intake and as such, the main meta-analysis is likely not affected by this. Second, the difference in median ferritin concentrations between the INMA and Generation R Studies was relatively large, which could be due to differences in socioeconomic position as indicated by the reported differences in education and smoking status of the participants. We do not expect these differences to have affected the reported associations, as we adjusted for factors related to socioeconomic position in our analyses. Serum ferritin concentrations were still mostly within the normal range, indicating a potential selection toward a healthier population. This could affect the generalizability of our findings. Third, serum ferritin was measured once during early pregnancy. Future studies with information on maternal serum ferritin concentrations at multiple time points during pregnancy would be helpful to observe whether patterns of serum ferritin concentrations throughout pregnancy may be more informative than measurements at a single time point. Fourth, we modeled the association of maternal serum ferritin concentrations with offspring DNA methylation linearly. However, it is possible that there might be a U-shaped relationship, as both low and high concentrations are associated with negative birth and offspring outcomes. Fifth, results are restricted to blood, but DNA methylation patterns may be different in other tissues. Blood is of particular interest, since hematological phenotypes are related to iron metabolism. However, in addition to blood, other tissues such as brain may also be relevant. Sixth, since the Illumina Chip Array only measures around 2% of the CpGs in the genome, it is possible that associations with DNA methylation levels at other, unmeasured CpGs exist. Last, the study participants are of European ancestry, meaning that the findings might not be generalizable to populations of a different ethnic background.

## Conclusions

Maternal early-pregnancy serum ferritin concentrations were associated with differential DNA methylation at two CpGs in *PRR23A* and one CpG in *PRSS22* in offspring. These associations partly persisted in children of older ages. Further studies are needed to confirm these findings and to uncover their role in the underlying mechanisms of the associations of maternal iron status and offspring health outcomes.

## Methods

**Fig. 2:**
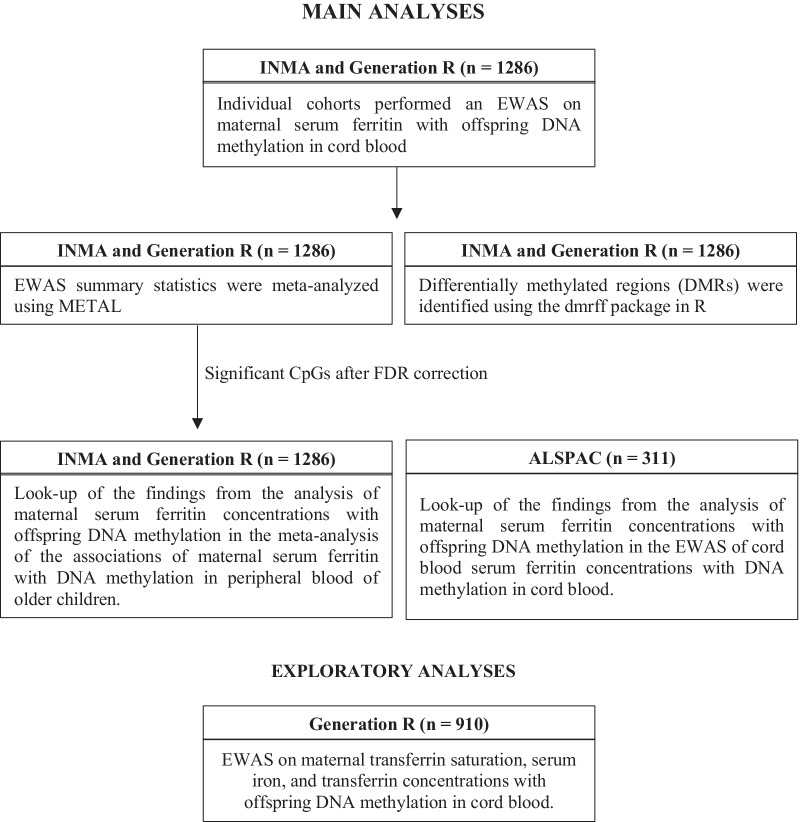
Overview of performed analyses. ALSPAC, Avon Longitudinal Study of Parents and Children. EWAS, epigenome-wide association study. INMA, INfancia y Medio Ambiente—(Environment and Childhood) Project

A full overview of the analyses performed is shown in Fig. 2.

### Participating cohorts

The meta-analysis examining the associations of maternal serum ferritin concentrations with cord blood DNA methylation was performed in the Generation R Study (*n* = 910) and the Proyecto Infancia y Medio Ambiente (INMA) Study (*n* = 376). An additional look-up of the findings from the primary analysis in an analysis of cord blood serum ferritin concentrations with cord blood DNA methylation was performed in the Avon Longitudinal Study of Parents and Children (ALSPAC). Cohort details are described in Additional file 1. Ethical approval and informed consent for all participants were obtained in the individual cohorts prior to data collection.

### Iron status assessment

Maternal venous whole blood samples were collected during early pregnancy in the Generation R and INMA Studies (median 12.8 (95% range 9.9, 17.0) and 13.2 (95% range 11.0, 17.2) weeks of gestation, respectively). The blood samples were collected in a non-fasting state at the Generation R Study and in a fasting state at the INMA Study. Serum ferritin reflects body iron stores and was defined as our primary exposure of interest. Within the Generation R Study only, transferrin saturation (TSAT), serum iron, and transferrin were also assessed and we predefined these markers of iron status as secondary exposures, since they are more prone to diurnal variations and are sensitive to recent intake of iron-containing food [38]. These markers provide additional information on the bioavailability of iron in the body. TSAT was calculated using serum iron and transferrin concentrations (TSAT [%] = (serum iron [µmol/L] * 100) / (transferrin [g/L] * 25.1)) to reflect the iron-bound part of the total iron binding capacity [38]. In the ALSPAC Study, serum ferritin was measured from cord blood samples (median 40.0 (95% range 36.0, 42.0) weeks of gestation).

### DNA methylation measurement

DNA from whole blood was bisulfite converted using the EZ-96 DNA Methylation Kit (Zymo Research Corporation, Irvine, CA, USA). Each cohort measured DNA methylation using the Infinium HumanMethylation450 BeadChip array (Illumina, San Diego, CA, USA), in cohort-specific laboratories, and each cohort conducted its own quality control and normalization of data, as described in Additional file 1. Outlying methylation beta values were excluded using the following method: Values < (25th percentile – 3 * interquartile range (3IQR)) and values > (75th percentile + 3IQR) were removed [39]. Probes on the *X* and *Y* chromosomes were excluded. Probes with low fluorescence in the array were removed using a detection p value cutoff specific to the individual’s cohort quality control. For all analyses, normalized, untransformed *β* values were used as outcomes.

### Covariates

Potential confounders were selected based on previous literature and were included if they were associated with both the exposure and the outcome in the literature [40, 41]. We selected maternal age at intake, educational level, pre-pregnancy body mass index, and smoking as potential confounders. In addition, we included gestational age at blood sampling as a precision variable for the exposure and child sex, blood cell subtypes, and batch as precision variables for the outcome. Maternal age at intake, pre-pregnancy weight, educational level, and smoking were obtained from questionnaires. Maternal height was measured at intake. Information on gestational age at birth, child sex, and birth weight was obtained from medical records. We took batch effects into account by including plate number as a covariate in the analyses in the Generation R Study and by using ComBat before running the association models in INMA [42]. We estimated the relative proportions of six white blood cell subtypes (CD4+ T lymphocytes, CD8+ T lymphocytes, natural killer cells, B lymphocytes, monocytes, and granulocytes) and nucleated red blood cells using a cord blood-specific reference for the cord blood DNA methylation analysis [43]. In the Generation R Study, we also tested the effect of adjusting for the first four principal components to correct for potential ethnic differences [44], as well as for maternal diet using a Mediterranean diet quality score [45], but neither of these covariates led to a > 10% median change in effect estimates and they were therefore not included in the main model. Cohort-specific details for covariate assessment are described in Additional file 1.

### Cohort-specific statistical analyses

Analyses were run in the individual cohorts according to a pre-defined analysis plan. We used robust linear regression models in an EWAS framework to analyze associations of maternal early-pregnancy serum ferritin concentrations (exposure) with single-CpG DNA methylation in cord blood (outcome). The analyses were performed in three models, and each cohort included the same covariates in each model. The first model was adjusted for gestational age at serum ferritin measurement, child sex, and batch by including these covariates in the model. The second model was additionally adjusted for maternal age at intake, educational level, pre-pregnancy body mass index (BMI), and smoking. The third model (main model) was adjusted for the same covariates as the second model, with additional adjustment for estimated cell-type proportions. The rlm function of the MASS R package was used to run the robust linear regression analyses. All analyses were performed using R version 3.4.3 [46].

### Meta-analyses

We performed a meta-analysis using the full output of the cohort-specific EWAS using inverse variance–weighted fixed-effects meta-analysis in METAL [47]. A total of 455,860 CpGs were included in the meta-analyses. To rule out potential human error, a second analyst (P.d.P-B.) performed an independent meta-analysis with the same methodology. Heterogeneity between studies was assessed using the I^2^ statistic. We used FDR correction for multiple testing, using the method by Benjamini and Hochberg [48].

We identified differentially methylated regions (DMRs) in relation to maternal early-pregnancy serum ferritin concentrations from the meta-analysis results with the dmrff package in R [49]. Candidate DMRs were defined as regions spanning at least two CpG sites with a maximum of 500 base pairs between consecutive sites with nominal EWAS *p* values < 0.05 and effect estimates with the same direction. Pairwise CpG site correlations were obtained from the EWAS summary statistics of each cohort. We calculated dmrff statistics for each candidate region accounting for the correlations. The dmrff statistics were meta-analyzed across the datasets [49]. DMRs were considered significant if the *p* value was < 1.1 × 10^−7^.

### Additional analyses

We examined whether associations of any CpGs identified might be mediated by gestational age at birth or by birth weight by additionally adjusting the main model for gestational age at birth and birth weight. As ferritin is also an acute phase protein, we performed a sensitivity analysis excluding all mothers with C-reactive protein (CRP) concentrations > 10 mg/L to remove participants with acute inflammation. In sensitivity analyses, we tested the effect of diet, as represented by the Mediterranean diet score, and the first genetic principal component (PC1) on the results for the significant CpGs from the main model. We performed additional meta-analyses additionally adjusting the main model for diet (*n* = 1208) or PC1 (*n* = 1200).

### Look-up analyses

We examined whether associations of any CpGs identified in cord blood persisted in the peripheral blood of older children, using the main model additionally adjusted for child age at measurement and using the Houseman reference for estimating six white blood cell-type proportions instead of the cord blood-specific reference used in the main models [50]. For this, we performed a meta-analysis on the associations of maternal early-pregnancy serum ferritin concentrations with single-CpG DNA methylation in early childhood (4- and 6-year-old children from INMA and the Generation R Study, respectively), and in late childhood (9- and 10-year-old children from INMA and the Generation R Study, respectively). In order to assess whether the effects on differential DNA methylation in offspring are specific to maternal serum ferritin concentrations, we examined whether CpGs differentially methylated in association with maternal serum ferritin concentrations were also differentially methylated in relation to cord blood serum ferritin in the ALSPAC Study. This analysis was adjusted for gestational age at serum ferritin measurement, maternal age at intake, educational level, pre-pregnancy body mass index, smoking, child sex, cell-type proportions, and batch (main model). CpGs were considered differentially methylated for these analyses if the p value was significant using Bonferroni correction (0.05/3 (number of CpGs)).

### Exploratory analyses of associations of maternal early-pregnancy TSAT, serum iron, and transferrin concentrations with DNA methylation at birth

Within the Generation R Study, we examined the associations of maternal early-pregnancy TSAT, serum iron, and transferrin concentrations with offspring DNA methylation, at single-CpG and differentially methylated region (DMR) level. In addition, we performed a look-up analysis of any identified CpGs from the primary serum ferritin meta-analysis in the associations of these additional maternal iron markers with offspring DNA methylation. CpGs were considered differentially methylated for these analyses if the p value was significant using Bonferroni correction (0.05/3 (number of CpGs)).

### Functional analyses

Using the CpGs that had a *p* value < 1.0 × 10^−5^ (18 CpGs) as input in the main serum ferritin analysis, we performed Kyoto Encyclopedia of Genes and Genomes (KEGG) pathway and gene ontology (GO) enrichment analyses using the missMethyl R package [51], and we assessed whether there was tissue-specific enrichment using the eFORGE online tool [52]. For the CpGs that were FDR-significant in the main serum ferritin analysis, we examined associations with expression levels of nearby genes by performing a look-up of these CpG sites in the HELIX *cis*-eQTM catalog [53]. We also performed a look-up in the UCSC genome browser to check if these CpGs were associated with regulatory regions [54]. Since the brain is a potentially relevant tissue in relation to iron-related phenotypes, we examined correlations between DNA methylation levels in blood and brain tissues at the FDR-significant CpG sites using the Blood Brain DNA Methylation Comparison Tool [21]. Next, for the CpGs that were FDR-significant in the main serum ferritin analysis and the exploratory analyses of the additional iron markers, we performed a look-up of the annotated genes in a mouse-knockout database [22]. Finally, for the CpGs that were FDR-significant in the main serum ferritin analysis, we performed a look-up in the results of previously published EWAS for neurodevelopmental outcomes including ADHD symptoms, autism spectrum disorder (ASD), and IQ [25–27].

## Supplementary Information

**Additional file 1.** Supplemental tables, cohort-specific methods, funding and acknowledgements.

**Additional file 2.** Supplemental tables.

## Data Availability

Full meta-analysis results will be made available through an open access database upon acceptance. Cohort-level data are available from the cohort senior authors upon reasonable request and may be subject to local regulations.

## Abbreviations

ALSPAC: Avon longitudinal study of parents and children
CRP: C-reactive protein
DMR: Differentially methylated region
GO: Gene ontology
INMA: Proyecto Infancia y Medio Ambiente
KEGG: Kyoto encyclopedia of genes and genomes
LBW: Low birth weight
SE: Standard error
SGA: Small for gestational age
TSAT: Transferrin saturation
