# Supplementary material for: Maternal iron status in early pregnancy and DNA methylation in offspring: an epigenome-wide meta-analysis
Source: Clin Epigenetics. 2022 May 3;14:59. doi: 10.1186/s13148-022-01276-w (PMC9066980; doi:10.1186/s13148-022-01276-w)
Supplement: Supplementary file 1 — Additional file 1. Supplemental tables, cohort-specific methods, funding and acknowledgements. [file 13148_2022_1276_MOESM1_ESM.docx]

**Supplementary Material**

**Maternal iron status in early pregnancy and DNA methylation in offspring: an epigenome-wide meta-analysis**

M.J. Taeubert^1,2^*, P. de Prado-Bert^3,4,5^*, M.L. Geurtsen^1,6^, G. Mancano^7,8^, M.J. Vermeulen^6^, I.K.M. Reiss^6^, J. Sunyer Deu^3,4,5,9^, G.C. Sharp^7,8,10^, J. Julvez^3,5,11^, M.U. Muckenthaler^2±^, J.F. Felix^1,6±^

1. The Generation R Study Group, Erasmus MC, University Medical Center Rotterdam, Rotterdam, the Netherlands.
2. Department of Pediatric Oncology, Hematology and Immunology, University Medical Center, Heidelberg, Germany.
3. ISGlobal, Barcelona, Spain.
4. Universitat Pompeu Fabra (UPF), Barcelona, Spain.
5. CIBER Epidemiología y Salud Pública (CIBERESP), Madrid, Spain.
6. Department of Pediatrics, Sophia’s Children’s Hospital, Erasmus MC, University Medical Center Rotterdam, Rotterdam, the Netherlands.
7. MRC Integrative Epidemiology Unit at the University of Bristol, Bristol, United Kingdom
8. Bristol Medical School Population Health Sciences, University of Bristol, Bristol, United Kingdom
9. IMIM (Hospital del Mar Medical Research Institute), Barcelona, Spain
10. School of Oral and Dental Sciences, University of Bristol, Bristol, United Kingdom
11. Institut d'Investigació Sanitària Pere Virgili, Hospital Universitari Sant Joan de Reus, Reus, Spain

* Joint first authors

^±^ Joint last authors

**Corresponding author**Janine Felix, The Generation R Study Group, Erasmus University Medical Center, PO Box 2040, 3000 CA Rotterdam, the Netherlands (j.felix@erasmusmc.nl). Telephone number: +31 10 7043405

**Content:**

**Table S1** Results of the meta-analysis of epigenome-wide association study results of maternal early-pregnancy serum ferritin concentrations and DNA methylation in cord blood with p values <1.0 x 10^-4^ – model 3

**Table S2** Results of the meta-analysis of epigenome-wide association study results of maternal early-pregnancy serum ferritin concentrations and DNA methylation in cord blood with p values <1.0 x 10^-4^ – model 1

**Table S3** Results of the meta-analysis of epigenome-wide association study results of maternal early-pregnancy serum ferritin concentrations and DNA methylation in cord blood with p values <1.0 x 10^-4^ – model 2

**Table S4** Associations of DNA methylation at the three CpGs identified in the meta-analysis of epigenome-wide association study results of maternal early-pregnancy serum ferritin concentrations and offspring DNA methylation, additionally adjusted for gestational age at birth (a) and for birth weight (b).

**Table S5** Results of the meta-analysis of epigenome-wide association study results of maternal early-pregnancy serum ferritin concentrations and DNA methylation in cord blood excluding mothers with C-reactive protein concentrations > 10 mg/L with p values <1.0 x 10^-4^

**Table S6** Associations of DNA methylation at the three CpGs identified in the meta-analysis of epigenome-wide association study results of maternal early-pregnancy serum ferritin concentrations and offspring DNA methylation, additionally adjusted for Mediterranean diet (a) and for the first genetic principal component (PC1) (b).

**Table S7** Maternal and child characteristics of the ALSPAC study

**Table S8** Results of the epigenome-wide association study results of maternal early-pregnancy transferrin concentrations and DNA methylation in cord blood with p values <1.0 x 10^-4^

**Table S9** Results of the epigenome-wide association study results of maternal early-pregnancy TSAT and DNA methylation in cord blood with p values <1.0 x 10^-4^

**Table S10** Results of the epigenome-wide association study results of maternal early-pregnancy serum iron and DNA methylation in cord blood with p values <1.0 x 10^-4^

**Table S11** Differentially Methylated Regions of the associations of maternal early-pregnancy TSAT concentrations with offspring DNA methylation in cord blood

**Table S12** Associations of DNA methylation at the three CpGs identified in the meta-analysis of epigenome-wide association study results of maternal early-pregnancy serum ferritin concentrations and cord blood DNA methylation in the EWAS of other maternal iron markers

**Table S13** Correlations of blood methylation levels with brain methylation levels at the three CpGs identified in the meta-analysis of epigenome-wide association study results of maternal early-pregnancy serum ferritin concentrations and cord blood DNA methylation

**Table S14** Associations of DNA methylation at the three CpGs identified in the meta-analysis of epigenome-wide association study results of maternal early-pregnancy serum ferritin concentrations and cord blood DNA methylation in the EWAS of neurodevelopmental outcomes: ADHD symptoms (a), autism spectrum disorder (ASD) (b), and IQ (c).

**Cohort-specific methods**

**Funding Support**

**Acknowledgements**

**Table S1.** Results of the meta-analysis of epigenome-wide association study results of maternal early-pregnancy serum ferritin concentrations and DNA methylation in cord blood with p values < 1.0 x 10^-4^ – model 3

*See separate Excel sheet*

**Table S2.** Results of the meta-analysis of epigenome-wide association study results of maternal early-pregnancy serum ferritin concentrations and DNA methylation in cord blood with p values <1.0 x 10^-4^ – model 1

*See separate Excel sheet*

**Table S3.** Results of the meta-analysis of epigenome-wide association study results of maternal early-pregnancy serum ferritin concentrations and DNA methylation in cord blood with p values <1.0 x 10^-4^ – model 2

*See separate Excel sheet*

**Table S4.** Associations of DNA methylation at the three CpGs identified in the meta-analysis of epigenome-wide association study results of maternal early-pregnancy serum ferritin concentrations and offspring DNA methylation, additionally adjusted for gestational age at birth (a) and for birth weight (b).

**a.**

| CpG | Chr | Position | Gene | Effect | SE | p value | I2 |
| --- | --- | --- | --- | --- | --- | --- | --- |
| cg02806645 | 3 | 138725153 | *PRR23A* | -2.6 x10^-4^ | 4.0 x10^-5^ | 1.3 x10^-10^ | 0 |
| cg06322988 | 3 | 138725189 | *PRR23A* | -1.1 x10^-4^ | 2.1 x10^-5^ | 8.4 x10^-8^ | 0 |
| cg04468817 | 16 | 2903392 | *PRSS22* | -1.2 x10^-4^ | 2.4 x10^-5^ | 1.9 x10^-7^ | 0 |

Chr, chromosome; SE, standard error; I2, I-square.

Effect estimates represent the difference in DNA methylation per 1 µg/L increase in maternal early-pregnancy serum ferritin concentrations. The model was adjusted for gestational age at serum ferritin measurement, maternal age at intake, educational level, pre-pregnancy body mass index, smoking, child sex, cell-type proportions, batch, and gestational age at birth.

**b.**

| CpG | Chr | Position | Gene | Effect | SE | p value | I2 |
| --- | --- | --- | --- | --- | --- | --- | --- |
| cg02806645 | 3 | 138725153 | *PRR23A* | -2.5 x10^-4^ | 4.0 x10^-5^ | 4.0 x10^-10^ | 0 |
| cg06322988 | 3 | 138725189 | *PRR23A* | -1.3 x10^-4^ | 2.4 x10^-5^ | 1.1 x10^-7^ | 0 |
| cg04468817 | 16 | 2903392 | *PRSS22* | -1.1 x10^-4^ | 2.1 x10^-5^ | 9.8 x10^-8^ | 24 |

Chr, chromosome; SE, standard error; I2, I-square.

Effect estimates represent the difference in DNA methylation per 1 µg/L increase in maternal early-pregnancy serum ferritin concentrations. The model was adjusted for gestational age at serum ferritin measurement, maternal age at intake, educational level, pre-pregnancy body mass index, smoking, child sex, cell-type proportions, batch, gestational age at birth, and birth weight.

**Table S5.** Results of the meta-analysis of epigenome-wide association study results of maternal early-pregnancy serum ferritin concentrations and DNA methylation in cord blood excluding mothers with C-reactive protein concentrations > 10 mg/L with p values <1.0 x 10^-4^

*See separate Excel sheet*

**Table S6.** Associations of DNA methylation at the three CpGs identified in the meta-analysis of epigenome-wide association study results of maternal early-pregnancy serum ferritin concentrations and offspring DNA methylation, additionally adjusted for Mediterranean diet (a) and for the first genetic principal component (PC1) (b).

**a.**

| CpG | Chr | Position | Gene | Effect | SE | p value | FDR | I2 |
| --- | --- | --- | --- | --- | --- | --- | --- | --- |
| cg02806645 | 3 | 138725153 | *PRR23A* | -2.3 × 10^−4^ | 4.1 × 10^−5^ | 3.5 × 10^−8^ | 1.7 × 10^−2^ | 0 |
| cg06322988 | 3 | 138725189 | *PRR23A* | -9.9 × 10^−5^ | 2.2 × 10^−5^ | 6.3 × 10^−6^ | 1.7 × 10^−1^ | 0 |
| cg04468817 | 16 | 2903392 | *PRSS22* | -1.2 × 10^−4^ | 2.4 × 10^−5^ | 8.5 × 10^−7^ | 1.5 × 10^−1^ | 38 |

Chr, chromosome; FDR, False Discovery Rate; SE, standard error; I2, I-square.

Effect estimates represent the difference in DNA methylation per 1 µg/L increase in maternal early-pregnancy serum ferritin concentrations. The model was adjusted for gestational age at serum ferritin measurement, maternal age at intake, educational level, pre-pregnancy body mass index, smoking, child sex, cell-type proportions, batch, and maternal Mediterranean diet score.

**b.**

| CpG | Chr | Position | Gene | Effect | SE | p value | FDR | I2 |
| --- | --- | --- | --- | --- | --- | --- | --- | --- |
| cg02806645 | 3 | 138725153 | *PRR23A* | -2.2 × 10^−4^ | 4.2 × 10^−5^ | 1.3 × 10^−7^ | 3.1 × 10^−2^ | 0 |
| cg06322988 | 3 | 138725189 | *PRR23A* | -1.0 × 10^−4^ | 2.2 × 10^−5^ | 4.3 × 10^−6^ | 1.8 × 10^−1^ | 0 |
| cg04468817 | 16 | 2903392 | *PRSS22* | -1.3 × 10^−4^ | 2.5 × 10^−5^ | 4.4 × 10^−7^ | 4.3 × 10^−2^ | 21 |

Chr, chromosome; FDR, False Discovery Rate; SE, standard error; I2, I-square.

Effect estimates represent the difference in DNA methylation per 1 µg/L increase in maternal early-pregnancy serum ferritin concentrations. The model was adjusted for gestational age at serum ferritin measurement, maternal age at intake, educational level, pre-pregnancy body mass index, smoking, child sex, cell-type proportions, batch, and the first genetic principal component (PC1).

**Table S7.** Maternal and child characteristics of the ALSPAC study

|  | ALSPAC Study  *n = 311* |
| --- | --- |
| Maternal characteristics |  |
| Age, years | 29.2 ± 4.4 |
| Pre-pregnancy body mass index, kg/m^2^ | 22.9 ± 3.6 |
| Gestational age at serum ferritin  measurement, weeks | 40.0 (36.0, 42.0) |
| Education, higher | 152 (48.9) |
| Continued smoking during pregnancy | 49 (15.8) |
| Serum ferritin, µg/L | 133.5 (37.1, 512.6) |
| Child characteristics |  |
| Sex, male | 159 (51.1) |
| Gestational age at birth, weeks | 40.0 (36.0, 42.0) |
|  |  |

Values are means ± SD, medians (95% range) or numbers of subjects (valid %).

**Table S8.** Results of the epigenome-wide association study results of maternal early-pregnancy transferrin concentrations and DNA methylation in cord blood with p values <1.0 x 10^-4^

*See separate Excel sheet*

**Table S9.** Results of the epigenome-wide association study results of maternal early-pregnancy TSAT and DNA methylation in cord blood with p values <1.0 x 10^-4^

*See separate Excel sheet*

**Table S10.** Results of the epigenome-wide association study results of maternal early-pregnancy serum iron and DNA methylation in cord blood with p values <1.0 x 10^-4^

*See separate Excel sheet*

**Table S11.** Differentially Methylated Regions of the associations of maternal early-pregnancy TSAT concentrations with offspring DNA methylation in cord blood

| DMR | CpGs | Effect | SE | p value | Nearest genes | Gene Group |
| --- | --- | --- | --- | --- | --- | --- |
| chr 16: 67225165-67225924 | cg05376405  cg09652746  cg03412237  cg02440872 | 1.0 x10^-4^ | 1.9 x10^-5^ | 3.7 x10^-8^ | *E2F4* | *TSS1500*  *TSS200* |

chr, chromosome; DMR, differentially methylated region; TSAT, transferrin saturation; TSS, transcription start site.

*DMRs were identified from the results of the epigenome-wide association studies of maternal early-pregnancy TSAT and DNA methylation in cord blood for individual CpGs, which used results from robust linear regression models that were adjusted for gestational age at TSAT measurement, maternal age at intake, educational level, pre-pregnancy body mass index, smoking, child sex, cell-type proportions, and batch.

**Table S12.** Associations of DNA methylation at the three CpGs identified in the meta-analysis of epigenome-wide association study results of maternal early-pregnancy serum ferritin concentrations and cord blood DNA methylation in the EWAS of other maternal iron markers

**a. Transferrin saturation**

| CpG | Chr | Position | Gene | Effect | SE | p value |
| --- | --- | --- | --- | --- | --- | --- |
| cg02806645 | 3 | 138725153 | *PRR23A* | -4.0 x10^-4^ | 2.1 x10^-4^ | 6.1 x10^-2^ |
| cg06322988 | 3 | 138725189 | *PRR23A* | -1.4 x10^-4^ | 1.1 x10^-4^ | 1.8 x10^-1^ |
| cg04468817 | 16 | 2903392 | *PRSS22* | -2.6 x10^-4^ | 1.4 x10^-4^ | 5.8 x10^-2^ |

Chr, chromosome; SE, standard error.

Effect estimates represent the difference in DNA methylation per % increase in maternal early-pregnancy transferrin saturation. The model was adjusted for gestational age at transferrin saturation measurement, maternal age at intake, educational level, pre-pregnancy body mass index, smoking, child sex, child age, cell-type proportions, and batch.

**b. Serum iron**

| CpG | Chr | Position | Gene | Effect | SE | p value |
| --- | --- | --- | --- | --- | --- | --- |
| cg02806645 | 3 | 138725153 | *PRR23A* | -3.1 x10^-4^ | 3.4 x10^-4^ | 3.7 x10^-1^ |
| cg06322988 | 3 | 138725189 | *PRR23A* | -9.3 x10^-5^ | 1.7 x10^-4^ | 5.8 x10^-1^ |
| cg04468817 | 16 | 2903392 | *PRSS22* | -2.6 x10^-4^ | 2.2 x10^-4^ | 2.4 x10^-1^ |

Chr, chromosome; SE, standard error.

Effect estimates represent the difference in DNA methylation per 1 μmol/L increase in maternal early-pregnancy serum iron concentrations. The model was adjusted for gestational age at serum iron measurement, maternal age at intake, educational level, pre-pregnancy body mass index, smoking, child sex, child age, cell-type proportions, and batch.

**c. Transferrin**

| CpG | Chr | Position | Gene | Effect | SE | p value |
| --- | --- | --- | --- | --- | --- | --- |
| cg02806645 | 3 | 138725153 | *PRR23A* | 1.9 x10^-2^ | 5.4 x10^-3^ | 5.3 x10^-4^ |
| cg06322988 | 3 | 138725189 | *PRR23A* | 8.9 x10^-3^ | 2.9 x10^-3^ | 2.0 x10^-3^ |
| cg04468817 | 16 | 2903392 | *PRSS22* | 8.1 x10^-3^ | 3.5 x10^-3^ | 2.0 x10^-2^ |

Chr, chromosome; SE, standard error.

Effect estimates represent the difference in DNA methylation per 1 g/L increase in maternal early-pregnancy transferrin concentrations. The model was adjusted for gestational age at transferrin measurement, maternal age at intake, educational level, pre-pregnancy body mass index, smoking, child sex, child age, cell-type proportions, and batch.

**Table S13.** Correlations of blood methylation levels with brain methylation levels at the three CpGs identified in the meta-analysis of epigenome-wide association study results of maternal early-pregnancy serum ferritin concentrations and cord blood DNA methylation

| CpG | Chr | Gene | Brain area | Correlation | n | p value |
| --- | --- | --- | --- | --- | --- | --- |
| cg02806645 | 3 | *PRR23A* | PFC | 0.56 | 74 | 2.6 x10^-7^ |
|  |  |  | EC | 0.53 | 71 | 1.7 x10^-6^ |
|  |  |  | STG | 0.52 | 75 | 1.9 x10^-6^ |
|  |  |  | CER | 0.42 | 71 | 3.2 x10^-4^ |
| cg06322988 | 3 | *PRR23A* | PFC | 0.54 | 74 | 6.7 x10^-7^ |
|  |  |  | EC | 0.51 | 71 | 6.6 x10^-6^ |
|  |  |  | STG | 0.57 | 75 | 9.0 x10^-8^ |
|  |  |  | CER | 0.56 | 71 | 3.0 x10^-7^ |
| cg04468817 | 16 | *PRSS22* | PFC | 0.25 | 74 | 2.9 x10^-2^ |
|  |  |  | EC | 0.46 | 71 | 5.6 x10^-5^ |
|  |  |  | STG | 0.47 | 75 | 2.4 x10^-5^ |
|  |  |  | CER | 0.26 | 71 | 2.7 x10^-2^ |

Chr, chromosome; CER, cerebellum; EC, entorhinal cortex; PFC, prefrontal cortex; STG, superior temporal gyrus.

**Table S14.** Associations of DNA methylation at the three CpGs identified in the meta-analysis of epigenome-wide association study results of maternal early-pregnancy serum ferritin concentrations and cord blood DNA methylation in the EWAS of neurodevelopmental outcomes: ADHD symptoms (a), autism spectrum disorder (ASD) (b), and IQ (c) (1-3).

**a. ADHD symptoms**

| CpG | Chromosome | Position | Gene | p value |
| --- | --- | --- | --- | --- |
| cg02806645 | 3 | 138725153 | *PRR23A* | 0.05 |
| cg06322988 | 3 | 138725189 | *PRR23A* | 0.18 |
| cg04468817 | 16 | 2903392 | *PRSS22* | 0.39 |

**b. Autism Spectrum Disorder (ASD)**

| CpG | Chromosome | Position | Gene | p value |
| --- | --- | --- | --- | --- |
| cg02806645 | 3 | 138725153 | *PRR23A* | 0.68 |
| cg06322988 | 3 | 138725189 | *PRR23A* | 0.20 |
| cg04468817 | 16 | 2903392 | *PRSS22* | 0.19 |

**c. IQ**

| CpG | Chromosome | Position | Gene | p value |
| --- | --- | --- | --- | --- |
| cg02806645 | 3 | 138725153 | *PRR23A* | 0.86 |
| cg06322988 | 3 | 138725189 | *PRR23A* | 0.20 |
| cg04468817 | 16 | 2903392 | *PRSS22* | 0.52 |

**Cohort-specific methods**

Avon Longitudinal Study of Parents and Children (ALSPAC)

The Avon Longitudinal Study of Parents and Children (ALSPAC) is a prospective pregnancy cohort study, which enrolled 14,541 pregnant women residing in Avon, United Kingdom who had expected delivery dates between April 1st, 1991 and December 31st, 1992. As described previously, detailed information has been collected on these participants and their offspring at regular intervals (4, 5). Details of all data collected for ALSPAC are available through a fully searchable data dictionary, which is publicly available: http://www.bris.ac.uk/alspac/researchers/data-access/data-dictionary/. As part of the Accessible Resources for Integrated Epigenomic Studies (ARIES, http://www.ariesepigenomics.org.uk/) project, DNA methylation was generated for 1,018 mother-offspring pairs from the ALSPAC cohort, using the Infinium HumanMethylation450 BeadChip array (Illumina Inc., San Diego, United States). ARIES participants were selected based on availability of DNA samples at two time points for the mother (antenatal and at follow-up when the offspring were adolescents). Ethical approval for the study was obtained from the ALSPAC Ethics and Law Committee and the Local Research Ethics Committees. Consent for biological samples has been collected in accordance with the Human Tissue Act (2004). Informed consent for the use of data collected via questionnaires and clinics was obtained from the participants following the recommendations of the ALSPAC Ethics and Law Committee at the time.

Iron status assessment

Ferritin was measured in cord heparin plasma at the ALSPAC laboratory using the DELFIA time resolved fluoroimmunoassay system. Ferritin assays were duplicated where possible and a coefficient of variation of approximately 4% was obtained.

DNA methylation

Methods for methylation measurements in ALSPAC have been described previously (6). Briefly, cord blood was collected according to standard procedures. DNA methylation assays and data pre-processing were performed at the University of Bristol as part of the ARIES project. DNA was extracted using standard protocol and was bisulfite-converted using the Zymo EZ DNA MethylationTM kit (Zymo, Irvine, CA). DNA methylation was then measured using the Infinium HM450 BeadChip assay (Illumina Inc, San Diego, CA), according to the standard protocol. Arrays were scanned using an Illumina iScan. An initial review of data quality was assessed using GenomeStudio (version 2011.1). A semi-random approach (sampling criteria were in place to ensure that all time points were represented on each array) was used to distribute ARIES samples across slides to minimize the possibility of potential confounding by batch. Data were normalised using the *meffil* R package (Min et al, 2018) using the functional normalisation approach. Blood cell type proportions was estimated using the “Salas” reference set in the ‘’FlowSorted.CordBlood.Combined.450K’’ Bioconductor package for cell type correction and normalised using *meffil* R package (7). QC steps included removal of sex chromosomes, SNPs and control probes with a detection p value over 0.05 for over 5% of samples. Methylation outliers were removed using the IQR*3 (Tukey) method.

Covariates

Maternal age at intake was determined by questionnaire at the time of recruitment. Maternal pre-pregnancy body mass index (BMI) was calculated from self-reported height and pre-pregnancy weight, which were collected by questionnaire during the first trimester of pregnancy. In ALSPAC, maternal education was defined based on the UK highest qualification achieved by the mothers. Maternal smoking during pregnancy was determined by questionnaire at the time of recruitment and defined in this study as no smoker, if mother was a never smoker or quit before second trimester, or otherwise a smoker. Newborn sex was obtained from obstetric records. Gestational age was calculated (in days) based on the date of the mother’s last menstrual period (LMP) when the mother was certain of this, but for uncertain LMPs and conflicts with clinical assessment the ultrasound assessment was used. Where maternal report and ultrasound assessment conflicted, an experienced obstetrician reviewed clinical records and made a best estimate. Gestational age in weeks was used in the analyses. We attempted to control for technical batch effects by generating 20 surrogate variables using the *SVA* R package (Leek et al. 2019, R package version 3.34.0) and including these directly in our models. Participants with non-white European ancestry were excluded from all analyses. Child ethnic group was derived from mother reports of her own and partners ethnic group during pregnancy.

**Generation R Study**

This study was embedded in the Generation R Study, a prospective population-based cohort from early fetal life onwards in Rotterdam, the Netherlands (8). The study design of the cohort has been described previously (8). The study has been approved by the Medical Ethical Committee of the Erasmus University Medical Center in Rotterdam (MEC 198.782/2001/31). Written informed consent was obtained for all participants (8). In total, 8,879 pregnant women were enrolled during pregnancy, of whom 6,159 had measurements of iron blood markers available. DNA methylation was measured in cord blood of a randomly selected European-ancestry subset of 1,396 participant. Out of these, 1,131 had measurements on early-pregnancy iron markers available. We excluded participants with missing covariates (*n* = 216), twin pregnancies and in case of multiple (non-twin) children per mother, we excluded one of each sibling pair, based on data completeness or, if equal, randomly (*n* = 5). The population for analysis of this study comprised 910 mother-newborn pairs.

Iron status assessment

Maternal non-fasting venous blood samples were collected during early pregnancy (median = 13.2 weeks (95% range 9.6 - 17.6 weeks)) (9). Serum ferritin was quantified using the electrochemiluminescence immunoassay “ECLIA” on the Cobas e411 analyzer (Roche, Almere, the Netherlands). TSAT was calculated using serum iron and transferrin levels (TSAT [%] = (serum iron [μmol/L] * 100) / (transferrin [g/L] * 25.1)) to reflect the iron-bound part of the total iron binding capacity (10). Serum iron was determined by the C502 module on the Cobas 8000 (Roche, Almere, the Netherlands) using a colorimetric assay. Transferrin was measured using the C502 module on the Cobas 8000 (Roche, Almere, the Netherlands), an immunoturbidimetric assay.

DNA methylation

DNA was extracted from cord blood using the salting-out method. Five hundred nanograms of DNA per sample underwent bisulfite conversion using the EZ-96 DNA Methylation kit (Shallow) (Zymo Research Corporation, Irvine, CA, USA). Samples were plated randomly onto 96-well plates. Samples were processed with the Illumina Infinium HumanMethylation450 (450 k) BeadChip (Illumina Inc., San Diego, CA, USA). Quality control of analyzed samples was performed using standardized criteria. Quality control and normalization of the array data was performed according to the Control Probe Adjustment and reduction of global CORrelation (CPACOR) workflow using R (11, 12). Probes that had a detection p value ≥ 1E^−16^ were set to missing per array. Additionally, the intensity values were quantile normalized for each of the six probe-type categories separately: type II red/green, type I methylated red/green, and type I unmethylated red/green. Beta values were calculated as proportion of methylated intensity value to the sum of (methylated and unmethylated intensities plus 100). Arrays with observed technical problems including failed bisulfite conversion, hybridization or extension, and arrays with a sex mismatch were removed from subsequent analyses. Only arrays with a call rate > 95% per sample were processed further. Probes on the X and Y chromosomes were excluded from the analyses. Outlying methylation beta values were excluded using the following method: values < (25th percentile – 3*interquartile range (3IQR)) and values > (75th percentile +3IQR) were removed (13).

Covariates

Information on maternal age, pre-pregnancy weight, educational level, and maternal smoking was obtained from questionnaires during pregnancy (8). Maternal height was measured at the intake visit without shoes. Pre-pregnancy BMI was calculated (self-reported pre-pregnancy weight in kilograms divided by height measured at enrolment in meters, squared). C-reactive protein (CRP) levels were measured in the same non-fasting venous blood samples that were used for the measurement of the iron markers, using an immunoturbidimetric assay on the Architect System (Abbot Diagnostics B.V., Hoofddorp, The Netherlands). Information on gestational age at birth, child sex, and birth weight was obtained from medical records. Plate number was included as a covariate in the analyses to adjust for batch effects. We estimated the relative proportions of six white blood cell subtypes (CD4+ T lymphocytes, CD8+ T lymphocytes, natural killer cells, B lymphocytes, monocytes, and granulocytes) and nucleated red blood cells using the cord blood-specific Salas reference (14).

**INMA**

The INMA—INfancia y Medio Ambiente— (Environment and Childhood) Project is a network of birth cohorts in Spain that aim to study the role of environmental pollutants in air, water and diet during pregnancy and early childhood in relation to child growth and development. In this study data on the cohort set up in Sabadell (Catalonia, Spain) between 2004 and 2008 was evaluated, further details explained elsewhere (15). The study has been approved by Ethical Committee of each participating center and written consent was obtained from participating parents. INMA Sabadell study has 622 children at birth, however cord blood DNA methylation was available for 391 children, and only 385 individuals had serum ferritin measurements. Considering complete cases (no missing in any of the covariates used) a final sample of 376 individuals was evaluated.

Iron status assessment

Maternal whole blood samples were collected during early pregnancy (median 13.2 weeks of gestation (95% CI: 11.0, 17.2 weeks)), by venipuncture under fasting conditions and stored between −70 and −80 °C until analysis. Maternal plasma ferritin concentrations were quantified in samples from Sabadell cohort by time-resolved fluorescence immunoassay (DELFIA Ferritin kit A069–101), at the Gipuzkoa Public Health Laboratory.

DNA methylation

Cord blood was extracted using the Chemagen kit (Perkin Elmer). DNA concentration was determined by NanoDrop spectrophotometer (Thermo Scientific) and with the Quant-iT PicoGreen dsDNA Assay Kit (Life Technologies). Methylation data was produced in two different laboratories as part of two different projects: in the Genome Analysis Facility of the University Medical Center Groningen (UMCG) in Holland, and in the Bellvitge Biomedical Research Institute (IDIBELL, Barcelona). Both laboratories used the recommended Illumina protocol for the Infinium 450k beadchip. Briefly, 500 nanograms of DNA was bisulfite-converted using the EZ 96-DNA methylation kit following the manufacturer’s standard protocol, and DNA methylation measured using the Illumina Infinium 450k beadchip. DNA methylation data were preprocessed using the *minfi* package (16). A series of steps were completed for quality control and data analysis. The first step was low quality sample removal. First, 2 samples with bad overall quality or with low detection p value according to the output of the *MethylAid* package were removed (17). Then, we removed 3 samples whose sex was wrongly predicted using *shinyMethyl* (18). Following guidelines of Lehne work (11), we increased the stringency of the detection p value threshold to 10-16 and we filtered 18 samples with a call rate lower than 98%. The second step was normalizing data with functional normalization. Correlation between SNP in replicates samples was checked and probes not measuring SNPs were discarded. 7,136 probes with a call rate lower than 95% were also removed. Probes in sex chromosomes, crosshibridizing or containing SNPs were flagged but not removed at this point. ComBat was applied to remove batch effect (19). Finally, duplicated samples were removed, prioritizing MeDALL samples over BREATHE samples. The final dataset consisted of 391 at age 0 years and 476,946 probes. For the current study we used European ancestry children.

Covariates

Information on maternal age, pre-pregnancy weight, education level and maternal smoking was collected by questionnaires (15). Maternal height was measured during the first prenatal visit. Pre-pregnancy BMI was calculated (self-reported pre-pregnancy weight in kilograms divided by height measured at enrolment in meters, squared). Maternal blood was collected at recruitment (mean ± SD, 13.4 ± 1.7 weeks of gestation). Serum CRP values were determined by turbidimetric assay using a Hitachi modular analyzer system (Roche Modular DPP, Hitachi Ltd, Tokyo, Japan) at the Laboratori de Referencia de Catalunya. The minimum detectable concentration of CRP was 0.2 mg/dL. For participants who had a CRP value below the detection limit (15.9%), we imputed a value of half of the detection limit. Gestational age at blood sampling was calculated based on last menstrual period (LMP) reported at recruitment and confirmed using estimates based on ultrasound examination in the 12th week of gestation. When the difference between the LMP reported at recruitment and estimated from the ultrasound was ≥ 7 days *(n* = 91; 16%), we estimated LMP using a quadratic regression formula defined by Westerway et al. (20). Birth weight was obtained from clinical records. We estimated the relative proportions of six white blood cell subtypes (CD4+ T lymphocytes, CD8+ T lymphocytes, natural killer cells, B lymphocytes, monocytes, and granulocytes) and nucleated red blood cells using a cord blood-specific reference (14).

**Funding Support**

Avon Longitudinal Study of Parents and Children (ALSPAC)

GM and GCS are members of the MRC Integrative Epidemiology Unit, which receives funds from the University of Bristol and United Kingdom Medical Research Council [MC_UU_00011/1, MC_UU_00011/5 and MC_UU_00011/6]. GCS’s contribution to this work is supported by the Medical Research Council [New Investigator Research Grant, MR/S009310/1]. GM and GCS’s contributions are supported by the European Joint Programming Initiative “A Healthy Diet for a Healthy Life” (JPI HDHL, NutriPROGRAM project, UK MRC MR/S036520/1]. The UK Medical Research Council and the Wellcome Trust (Grant ref: 217065/Z/19/Z) and the University of Bristol provide core support for ALSPAC. A comprehensive list of grants funding is available on the ALSPAC website (http://www.bristol.ac.uk/alspac/external/documents/grant-acknowledgements.pdf). The Accessible Resource for Integrated Epigenomics Studies (ARIES) which generated large scale methylation data was funded by the UK Biotechnology and Biological Sciences Research Council (BB/I025751/1 and BB/I025263/1). Additional epigenetic profiling on the ALSPAC cohort was supported by the UK Medical Research Council Integrative Epidemiology Unit and the University of Bristol (MC_UU_12013_1, MC_UU_12013_2, MC_UU_12013_5 and MC_UU_12013_8), the United States National Institute of Health (5RO1AI121226-02) and National Institute of Child and Human Development grant (R01HD068437). The funders had no role in study design, data collection and analysis, decision to publish, or preparation of the manuscript. This publication is the work of the authors and Gemma Sharp will serve as guarantors for the contents of this paper. The views expressed in this paper are those of the authors and not necessarily any funders. The funders had no influence on the content of the paper.

Generation R Study

The general design of the Generation R Study is made possible by financial support from the Erasmus Medical Center, Rotterdam; the Erasmus University Rotterdam; the Netherlands Organization for Health Research and Development; and the Ministry of Health, Welfare, and Sport. The EWAS data was funded by a grant from the Netherlands Genomics Initiative (NGI)/Netherlands Organisation for Scientific Research (NWO), Netherlands Consortium for Healthy Aging (NCHA; project nr. 050-060-810), by funds from the Genetic Laboratory of the Department of Internal Medicine, Erasmus MC, and by a grant from the National Institute of Child and Human Development (R01HD068437). This project received funding from the European Union’s Horizon 2020 research and innovation programme under grant agreement numbers 733206 (LifeCycle), 874739 (LongITools) and 824989 (EUCAN-Connect) and from the European Joint Programming Initiative “A Healthy Diet for a Healthy Life” (JPI HDHL, NutriPROGRAM project, ZonMw the Netherlands no.529051022 and PREcisE project, ZonMw the Netherlands no.529051023). MUM acknowledges funding from German Academic Exchange Service (DAAD) and the Dietmar Hopp Stiftung.

INMA

Main funding of the epigenetic studies in INMA were grants from Instituto de Salud Carlos III (Red INMA G03/176, CB06/02/0041), Spanish Ministry of Health (FIS-PI04/1436, FIS-PI08/1151 including FEDER funds, FIS-PI11/00610, FIS-FEDER-PI06/0867, FIS-FEDER-PI03-1615, MS13/00054, CP18/00018), Generalitat de Catalunya-CIRIT 1999SGR 00241, Fundació La Marató de TV3 (090430), EU Commission (261357-MeDALL: Mechanisms of the Development of ALLergy), and European Research Council (268479-BREATHE: BRain dEvelopment and Air polluTion ultrafine particles in scHool childrEn). J.J. holds a Miguel Servet-II contract (grants CPII19/00015) awarded by the Instituto de Salud Carlos III (Co-funded by the European Social Fund “Investing in your future”).

**Acknowledgements**

**Avon Longitudinal Study of Parents and Children (ALSPAC)**

We are extremely grateful to all the families who took part in this study, the midwives for their help in recruiting them, and the whole ALSPAC team, which includes interviewers, computer and laboratory technicians, clerical workers, research scientists, volunteers, managers, receptionists, and nurses. Please note that the ALSPAC study website (http://www.bristol.ac.uk/alspac/researchers/our-data/) contains details of all the data that is available through a fully searchable data dictionary and variable search tool.

**Generation R Study**

The Generation R Study is conducted by the Erasmus Medical Center in close collaboration with the School of Law and Faculty of Social Sciences of the Erasmus University Rotterdam; the Municipal Health Service Rotterdam area, Rotterdam; the Rotterdam Homecare Foundation, Rotterdam; and the Stichting Trombosedienst & Artsenlaboratorium Rijnmond (STAR-MDC), Rotterdam. We gratefully acknowledge the contribution of children and parents, general practitioners, hospitals, midwives, and pharmacies in Rotterdam. The study protocol was approved by the Medical Ethical Committee of the Erasmus Medical Centre, Rotterdam. Written informed consent was obtained for all participants. The generation and management of the Illumina 450K methylation array data (EWAS data) for the Generation R Study was executed by the Human Genotyping Facility of the Genetic Laboratory of the Department of Internal Medicine, Erasmus MC, the Netherlands. We thank Mr. Michael Verbiest, Ms. Mila Jhamai, Ms. Sarah Higgins, Mr. Marijn Verkerk, and Dr. Lisette Stolk for their help in creating the EWAS database. We thank Dr. A. Teumer for his work on the quality control and normalization scripts.

**INMA**

INMA researchers would like to thank all the participants for their generous collaboration. A full roster of the INMA Project Investigators can be found at http://www.proyectoinma.org/presentacioninma/listado-investigadores/en_listado-investigadores.html. We acknowledge support from the Spanish Ministry of Science and Innovation through the “Centro de Excelencia Severo Ochoa 2019-2023” Program [CEX2018-000806-S], and support from the Generalitat de Catalunya through the CERCA Program. J.J. holds Miguel Servet-II contract (CPII19/00015) awarded by the Instituto de Salud Carlos III (Co-funded by European Social Fund “Investing in your future”).

# References

1. Neumann A, Walton E, Alemany S, Cecil C, Gonzalez JR, Jima DD, et al. Association between DNA methylation and ADHD symptoms from birth to school age: a prospective meta-analysis. Transl Psychiatry. 2020;10(1):398.

2. Andrews SV, Sheppard B, Windham GC, Schieve LA, Schendel DE, Croen LA, et al. Case-control meta-analysis of blood DNA methylation and autism spectrum disorder. Mol Autism. 2018;9:40.

3. Caramaschi D, Neumann A, Cardenas A, Tindula G, Alemany S, Zillich L, et al. Meta-analysis of epigenome-wide associations between DNA methylation at birth and childhood cognitive skills. Mol Psychiatry. 2022.

4. Boyd A, Golding J, Macleod J, Lawlor DA, Fraser A, Henderson J, et al. Cohort Profile: the 'children of the 90s'--the index offspring of the Avon Longitudinal Study of Parents and Children. Int J Epidemiol. 2013;42(1):111-27.

5. Fraser A, Macdonald-Wallis C, Tilling K, Boyd A, Golding J, Davey Smith G, et al. Cohort Profile: the Avon Longitudinal Study of Parents and Children: ALSPAC mothers cohort. Int J Epidemiol. 2013;42(1):97-110.

6. Relton CL, Gaunt T, McArdle W, Ho K, Duggirala A, Shihab H, et al. Data Resource Profile: Accessible Resource for Integrated Epigenomic Studies (ARIES). Int J Epidemiol. 2015;44(4):1181-90.

7. Min JL, Hemani G, Davey Smith G, Relton C, Suderman M. Meffil: efficient normalization and analysis of very large DNA methylation datasets. Bioinformatics. 2018;34(23):3983-9.

8. Kooijman MN, Kruithof CJ, van Duijn CM, Duijts L, Franco OH, van IMH, et al. The Generation R Study: design and cohort update 2017. Eur J Epidemiol. 2016;31(12):1243-64.

9. Jaddoe VW, Bakker R, van Duijn CM, van der Heijden AJ, Lindemans J, Mackenbach JP, et al. The Generation R Study Biobank: a resource for epidemiological studies in children and their parents. Eur J Epidemiol. 2007;22(12):917-23.

10. Elsayed ME, Sharif MU, Stack AG. Transferrin Saturation: A Body Iron Biomarker. Adv Clin Chem. 2016;75:71-97.

11. Lehne B, Drong AW, Loh M, Zhang W, Scott WR, Tan ST, et al. A coherent approach for analysis of the Illumina HumanMethylation450 BeadChip improves data quality and performance in epigenome-wide association studies. Genome Biol. 2015;16:37.

12. Team RC. R: A language and environment for statistical computing. Vienna: R Foundation for Statistical Computing. 2014.

13. Tukey J. Exploratory data analysis. Reading: Addison-Wesley. 1997.

14. Gervin K, Salas LA, Bakulski KM, van Zelm MC, Koestler DC, Wiencke JK, et al. Systematic evaluation and validation of reference and library selection methods for deconvolution of cord blood DNA methylation data. Clin Epigenetics. 2019;11(1):125.

15. Guxens M, Ballester F, Espada M, Fernandez MF, Grimalt JO, Ibarluzea J, et al. Cohort Profile: the INMA--INfancia y Medio Ambiente--(Environment and Childhood) Project. Int J Epidemiol. 2012;41(4):930-40.

16. Aryee MJ, Jaffe AE, Corrada-Bravo H, Ladd-Acosta C, Feinberg AP, Hansen KD, et al. Minfi: a flexible and comprehensive Bioconductor package for the analysis of Infinium DNA methylation microarrays. Bioinformatics. 2014;30(10):1363-9.

17. van Iterson M, Tobi EW, Slieker RC, den Hollander W, Luijk R, Slagboom PE, et al. MethylAid: visual and interactive quality control of large Illumina 450k datasets. Bioinformatics. 2014;30(23):3435-7.

18. Fortin JP, Fertig E, Hansen K. shinyMethyl: interactive quality control of Illumina 450k DNA methylation arrays in R. F1000Res. 2014;3:175.

19. Johnson WE, Li C, Rabinovic A. Adjusting batch effects in microarray expression data using empirical Bayes methods. Biostatistics. 2007;8(1):118-27.

20. Westerway SC, Davison A, Cowell S. Ultrasonic fetal measurements: new Australian standards for the new millennium. Aust N Z J Obstet Gynaecol. 2000;40(3):297-302.
